# Peri-Insular Hemispherotomy: A Systematic Review and Institutional Experience

**DOI:** 10.1159/000529098

**Published:** 2023-01-13

**Authors:** Charles F. Yates, Stephen Malone, Kate Riney, Ubaid Shah, Martin J. Wood

**Affiliations:** ^a^Department of Neurosurgery, Queensland Children's Hospital, Brisbane, Queensland, Australia; ^b^Royal Brisbane and Women's Hospital, Brisbane, Queensland, Australia; ^c^School of Medicine, University of Queensland, Brisbane, Queensland, Australia; ^d^Neurosciences Unit, Queensland Children's Hospital, Brisbane, Queensland, Australia; ^e^Centre for Advance Imaging, University of Queensland, Brisbane, Queensland, Australia

**Keywords:** Peri-insular hemispherotomy, Epilepsy, Paediatric patient

## Abstract

**Introduction:**

Peri-insular hemispherotomy (PIH) is a hemispheric separation technique under the broader hemispherotomy group, a surgical treatment for patients with intractable epilepsy. Hemispherotomy techniques such as the PIH, vertical parasagittal hemispherotomy (VPH), and modified-lateral hemispherotomy are commonly assessed together, despite significant differences in anatomical approach and patient selection. We aim to describe patient selection, outcomes, and complications of PIH in its own right.

**Methods:**

A systematic review of the literature, in accordance with the Preferred Reporting Items of Systematic Reviews and Meta-Analyses (PRISMA) guidelines, was conducted, with searches of the PubMed and Embase databases. A local series including patients receiving PIH and followed up at the Queensland Children's Hospital between 2014 and 2020 was included.

**Results:**

Systematic review of the literature identified 393 patients from 13 eligible studies. Engel class 1 outcomes occurred in 82.4% of patients, while 8.6% developed post-operative hydrocephalus. Hydrocephalus was most common in the youngest patient cohorts. Developmental pathology was present in 114 (40.8%) patients, who had fewer Engel 1 outcomes compared to those with acquired pathology (69.1% vs. 83.7%, *p* = 0.0167). The local series included 13 patients, 11/13 (84.6%) had Engel class 1 seizure outcomes. Post-operative hydrocephalus occurred in 2 patients (15.4%), and 10/13 (76.9%) patients had worsened neurological deficit.

**Conclusion:**

PIH delivers Engel 1 outcomes for over 4 in 5 patients selected for this procedure, greater than described in combined hemispherectomy analyses. It is an effective technique in patients with developmental and acquired pathologies, despite general preference of VPH in this patient group. Finally, very young patients may have significant seizure and cognitive benefits from PIH; however, hydrocephalus is most common in this group warranting careful risk-benefit assessment. This review delivers a dedicated PIH outcomes analysis to inform clinical and patient decision-making.

## Introduction

Hemispheric separation has evolved since it was first used by Dandy [1] in 1928 for glioma resection and for intractable epilepsy by McKenzie [2] in 1938 (anatomic hemispherectomy). Functional hemispherectomy was introduced in the 1970s, reducing the degree of tissue resection to the central and temporal regions, disconnecting the remainder [3]. Finally, hemispherotomy was described in the 1990s with vertical parasagittal (VPH) and lateral approaches to further reduce the degree of resection [4, 5, 6]. Hemispherotomy is superior to anatomical hemispherectomy with regard to complications such as hydrocephalus and superficial cerebral haemosiderosis [4, 5, 7, 8] and equally effective in seizure freedom outcomes [7, 9, 10]. Peri-insular hemispherotomy (PIH) is a type of lateral hemispherotomy involving resection of peri-insular tissue or the temporal lobe [5, 11, 12]. Patient selection normally requires medically refractory seizures localized to one hemisphere, resulting from pathology not amenable to focal resection [5].

The goals of hemispheric separation procedures are common, achieving seizure control, epileptic encephalopathy cessation, and improved quality of life. However, these are achieved via different anatomical courses and degrees of tissue resection. VPH is commonly used for patients with gross distortion of cortical anatomy with developmental pathologies [13, 14], a patient group which generally experiences poorer outcomes [9, 15]. PIH involves resection of the insula cortex whereas VPH disconnects the insula from a superior approach. Residual insula connection may elicit seizure recurrence [12, 13]. The modified lateral hemispherotomy proposed by Cook et al. [4] includes middle cerebral artery sacrifice, absent in other hemispherotomy techniques. Finally, some evidence suggests seizure outcomes may not be equal between techniques, and different approaches to disconnection risk higher rates of incomplete disconnection and therefore seizure recurrence [14, 16, 17].

Despite these differences, the assessment of particular techniques such as PIH is limited to combined hemispherotomy analyses, or small PIH case series. Key issues such as outcomes and complications may be disparate given anatomical approach and patient selection between techniques is distinct. Therefore, this study aimed to systematically review the literature on PIH to inform decision-making for the use of PIH specifically.

## Methods and Materials

Following approval by the Human Research Ethics Committee of Queensland Children's Hospital, a retrospective chart review was completed for patients receiving PIH, at the Queensland Children's Hospital, alongside a systematic review of the literature.

### Systematic Review

Search of the PubMed and Embase databases was conducted. Search terms “Hemispherectomy”, “Hemispherotomy”, “Functional”, “Lateral”, “Peri-insular”, and “PIH” were used with Boolean operators “AND,” “OR.” The term “hemispherectomy” was included to account for traditional use of this term describing multiple hemispheric separation techniques. Reference lists of all eligible manuscripts were screened for further literature. Inclusion criteria involved PIH surgical technique, seizure outcome evaluation with complications, >6-month follow-up, >10 patients, and English language. Review articles and inadequate data availability were reasons for exclusion. Where multiple studies were published from the same institution, the single study with the most comprehensive results was included only to avoid risk of repeat analysis. This review was conducted in compliance with the Preferred Reporting Items for Systematic Reviews and Meta-Analyses (PRISMA) statement (see online suppl. Table at www.karger.com/doi/10.1159/000529098) [18]. The search was dated from inception to December 1st, 2021. Study quality was assessed according to the Oxford Centre for Evidence-Based Medicine: Levels of Evidence (March 2009) criteria. Where averages are calculated, these represented weighted averages accounting for study sample size. Where descriptive statistics included averages, these were weighted by patient number as a proportion of total patients in the systematic review.

### Case Series

A retrospective review of the neurosurgical database for all patients receiving PIH between 2015 and 2020 and followed at the Queensland Children's Hospital (QCH), Australia, was conducted. Inclusion criteria were defined as patients 0–18 years old, receiving PIH by a single neurosurgeon (MW), and followed at QCH. Follow-up <6 months and age over 18 years at time of surgery were excluded. The epilepsy type prior to surgery was categorized per the International League Against Epilepsy classifications (2017). The primary outcome was the presence or absence of seizures, as described by the Engel classification [19], and secondary outcome, an assessment of motor sensory function compared with the pre-surgical state. Neurocognitive outcomes were not explored in this review.

Complications were defined as any deviation from the expected treatment course. The surgical technique used in this centre most closely represented that described by Villemure and Mascott [11].

Underlying pathology was dichotomized into developmental and acquired types, developmental included multi-lobar cortical dysplasia, hemimegalencephaly, and Sturge-Weber syndrome. Acquired pathologies included vascular insult (ischaemic or haemorrhagic), hemiconvulsion-hemiplegia epilepsy syndrome, Rasmussen's encephalitis (RE), or trauma.

## Results

### Systematic Review

**Fig. 1:**
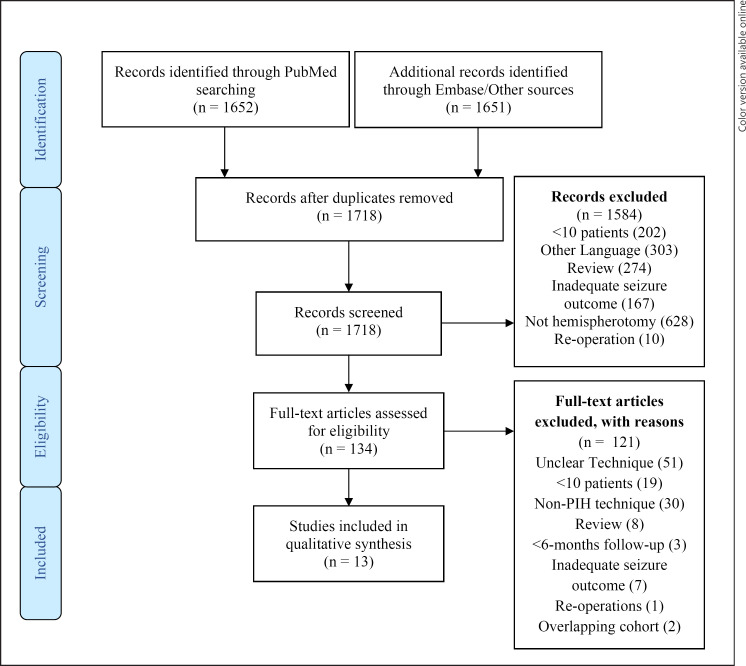
Preferred Reporting for Systematic Reviews and Meta-Analyses (PRISMA) diagram, outlining the review strategy [18].

The initial literature search yielded 1,652 and 1,651 articles from PubMed and Embase, respectively, from which 1,718 were included after removing duplications. Following abstract and title screening, 1,584 studies were excluded (shown in Fig. 1). 134 full-text manuscripts were reviewed for eligibility, from which 121 were excluded. Patient data and outcomes were extracted from 13 eligible articles [5, 7, 20, 21, 22, 23, 24, 25, 26, 27, 28, 29, 30]. A total of 393 patients receiving PIH were included in the systematic review, with a range of 11–83 patients (Table 1). 11/13 studies assessed paediatric populations, two included paediatric and adult patients. 61.1% of patients were male. The average age at surgery was 6.8 years, with a 4.0-year average delay to surgery from epilepsy diagnosis. Intellectual impairment was present in 84.0% of patients. Where aetiology was available (11/13 studies), 197/333 (59.2%) patients had acquired aetiologies, while 136/333 (40.8%) had developmental aetiologies (Table 2).

Engel 1 seizure outcomes were recorded in 82.4% of patients in the 11/13 studies recording Engel 1 outcomes. Two studies combined Engel 1 and 2 outcomes. Overall, 88.4% of patients had an Engel 1/2 outcome. Post-operative hydrocephalus necessitating ventriculoperitoneal shunt (VPS) insertion occurred in 8.6% of cases. 2/279 patients died, each in separate studies. Neurological deficit (hemiplegia and hemianopia) was worsened in 28/68 (41.1%) patients in which this was recorded. Finally, studies with a greater than 50% proportion of patients with developmental pathology recorded Engel class 1 outcomes in 69.1% of patients (weighted average) [7, 20, 23, 25, 27], compared to 83.7% in the remaining studies of the systematic review (*p* = 0.0167).

### Case Series

PIH was performed in 13 patients with more than 6 months follow-up. These included 8 male and 5 female patients, with an average age at surgery of 5.9 years, and 3.7 years average delay from diagnosis of epilepsy to surgery. In one infant, an initial delay to surgery was a parental choice, despite a significant encephalopathy and AED load. Intellectual impairment was present in 10/13 (76.9%) patients, but 2 patients were too young to be formally tested. Mean follow-up duration was 1.7 years.

Acquired ischaemic aetiology was present in 4/13 (30.8%) patients, Sturge-Weber syndrome in 2 patients (15.4%), hemiconvulsion-hemiplegia epilepsy syndrome in 2 patients (15.4%), RE in 1 patient (7.7%), and diffuse cortical malformations (cortical dysplasia and hemimegalencephaly) in 3 patients (23.1%). Focal seizures alone were observed in 7/13 patients (53.8%) while 6/13 (46.2%) patients had focal to bilateral tonic-clonic, absence/atypical absence seizures, or epileptic spasms. Seizure frequency was recorded as daily or multiple daily in 10/13 patients (76.9%) and weekly or less in 3/13 patients (23.1%). Four patients had hemispheric cortical malformations, of which all were hospital/ICU care dependent due to multiple daily seizures, with three having had failed focal surgical resections prior.

Following surgery, Engel 1 outcomes were recorded in 11/13 (84.6%) patients (100% Engel 1 or 2). AEDs were ceased in 7/13 (53.8%) patients. A further 4/13 (30.8%) patients were having AEDs weaned at follow-up but were early in the follow-up phase (mean follow-up of 9 months). One patient (patient 3) remained on sulthiame for continuous spike and wave during sleep in the contralateral hemisphere, without clinical seizures, and which was improving. One patient had intermittent seizures related to febrile illness.

Complications were experienced in 5/13 (38.5%) patients. Two (15.4%) of these were hydrocephalus requiring VPS insertion, and one patient developed a subdural hygroma post-operatively, necessitating a subdural-peritoneal shunt. A third patient had syndrome of inappropriate antidiuretic hormone causing hyponatraemic seizures. One patient had ongoing seizures secondary to a residual basal frontal connection. This patient returned to theatre for disconnection, after which seizure burden was significantly reduced. The outcome for this patient (Engel 2) is recorded after the second procedure.

Increased neurological deficit occurred in 10/13 (76.9%) patients. Hemiplegia worsened in 10/13 (76.9%) patients, two patients had hemianopia as a result of surgery, one patient (with dominant lobe surgery for RE) had dysphasia, and one patient had oculomotor nerve palsy.

## Discussion

PIH is a recent evolution of hemispheric separation techniques with a lateral approach and minimal tissue resection. Despite distinct differences in anatomical course and patient selection between hemispherectomy techniques, evidence on outcomes and complications are limited to combined technique assessments, or smaller case series. We present an analysis on a 393-patient cohort from a systematic review and institutional case series of patients undergoing PIH.

Patients suffering intractable epilepsy may derive significant benefit with PIH, as observed in our systematic review (Table 1, 82.4% with an Engel 1 outcome, 88.4% Engel 1/2). Comparable outcomes were recorded in the institutional series (Table 3, 84.6% Engel class 1, 100% class 1/2). Seizure freedom following hemispheric separation in combined analyses is described in 73–76% of patients [9, 15, 16, 29]. Seizure outcomes following PIH compared to other techniques are debated, some describing no difference in outcomes [16, 31, 32], others describing poorer outcomes [14]; however, these observations are predominantly made in very small patient cohorts. Here, we observe higher Engel 1 outcome rates compared with combined analyses, which may be explained by insular resection as sparing of the insula is associated with residual connection and poorer seizure outcomes [12, 27]. This is demonstrated in one class 4 study from the systematic review, showing higher rates of complete disconnection with PIH compared to an alternative hemispherotomy technique [26]. Another class 4 analysis observed 80% Engel 1 outcomes in their PIH cohort, compared with 62.5% in the combined assessment (PIH, VPH) [29]. Finally, the general preference of alternative hemispherectomy techniques to PIH for developmental pathology may underlie some outcome differences [9, 16].

Developmental pathology has consistently been associated with poorer outcome following hemispherotomy [4, 8, 13, 16]. In our systematic review, 40.8% of patients had developmental pathology (Table 2). In a weighted subgroup analysis of studies with greater than 50% patients with developmental pathology, 69.1% of patients had Engel 1 outcomes [7, 20, 23, 25, 27], compared to 83.7% Engel 1 outcomes in the remaining studies of the systematic review (*p* = 0.0167). Villemure and Daniel [5] assessed a large hemispherotomy cohort, finding children with perinatal ischaemic insult and RE were more likely to have Engel 1 outcomes (93% and 90%), compared to patients with developmental malformations (80%). Similarly, Hu et al. [9] found in their meta-analysis of combined techniques that developmental aetiologies had a significantly lower likelihood of seizure freedom post-operatively regardless of technique, as supported by others [4, 21, 33]. One hypothesis for this is an increased risk of incomplete disconnection, due to distortion of anatomical landmarks with developmental malformations [17, 33]. Furthermore, patients with developmental abnormalities are more likely to have contralateral hemisphere abnormalities [9], which are challenging to appreciate on pre-operative imaging and are a risk for poor seizure outcomes [16, 34]. Generally VPH is preferred for patients with developmental pathology; however, our results show similar seizure outcomes in this patient group using PIH (Table 2). In the original paper describing the VPH technique, Delalande et al. [13] recorded 74% of patients having an Engel 1 outcome. In their series, 30/84 (35.7%) children had dysplastic pathology, a similar portion to the current systematic review (40.8%). Interestingly, one large class 3 study of 84 patients undergoing PIH did not observe any significant difference in Engel outcome between developmental and acquired pathology groups (86.9%, 83.3%, respectively) [28]. These insights suggest surgeon preference and experience may be more important than technique differences for patients with developmental pathology.

The duration of epilepsy may also negatively impact seizure outcomes post-PIH [9, 35]; however, the balance of delaying against the risk of complication, neurological deficits, and reoperation in younger patients is a key issue [9, 31]. One class 4 study in the systematic review demonstrates this in 14 infants under 1-year old, finding 78.6% achieved an Engel 1 outcome [23]; however, 28.6% required VPS insertion and 1 patient died. Delaying surgery until possible development of a hemiparesis makes surgical intervention more favourable; however, epilepsy duration and persistent seizures may significantly impact cognitive function [36]. Kadish et al. [37] evaluated 25 children under 3 years undergoing hemispherotomy, concluding that delay to surgery from first seizure was the only modifiable factor predictive of worse cognitive outcome. Another class 4 study evaluated 11 patients with RE, observing significantly greater cognitive improvements in children with a shorter duration of epilepsy [24]. This is supported by an early functional MRI study which demonstrated transfer of sensorimotor function to the contralateral hemisphere following hemispherectomy [38]. Epilepsy duration at our institution was 3.7 years, similar to that in the systematic review (4.0 years). Our series included three patients operated on in the first year of life, all with diffuse hemispheric malformations. These patients were hospital/intensive care bound, and PIH served as a life-saving procedure. They had an Engel 1 outcome of 66.7% (Engel 1 and 2 100%), and VP shunt requirement in 1 (33.3%), but no patient required reoperation. The delay to hemispherotomy was related to previous failed localized surgeries but also parental requests for alternative treatments in 1 patient. Our experience has led to younger patients being considered for PIH, and surgery expedited acknowledging the increased surgical risks in the younger patients. Finally, weaning of AEDs is likely to impact cognition, and our series demonstrated 7/13 patients were AED free at follow-up, with 4/13 still weaning at follow-up.

Another consideration in the pre-operative workup for possible PIH is the impact of electroencephalogram (EEG) lateralization on post-operative seizure outcomes, which is widely debated [8, 9, 25, 37]. Some evidence supports contralateral EEG activity predicting poorer seizure outcomes [9, 21, 25, 28, 39], while others have suggested this does not affect seizure outcomes [8, 16]. Our systematic review identified two class 3 studies assessing this variable. One large cohort study demonstrated contralateral EEG and PET abnormalities predicted poorer outcomes on univariate analysis; however, only PET activity was significant on multivariate analysis. The second similarly found that contralateral EEG abnormalities had no significant effect on seizure outcomes [22]. The authors cite EEG measurement inaccuracies such as reduced amplitude in an atrophied hemisphere that distort contralateral hemisphere ictal recordings [40]. These results are repeated in a similar large hemispherotomy cohort [16]. Our institutional experience included 6/13 (46.2%) patients with focal seizures evolving to bilateral hemispheric ictal activity, of which all but one are seizure free, supporting the concept that patients with bilateral hemisphere ictal activity can still achieve good outcomes with PIH in the paediatric age. Finally, bilateral hemispheric involvement is more likely in developmental pathology, which may confound lateralization as a predictor of outcome.

Post-operative neurological deficits can hold significant morbidity for patients and their families and are a key consideration. Common deficits following PIH include contralateral hemiplegia, homonymous hemianopia, and potential language deficits (if operating on the language dominant hemisphere) [5]. Patients often recover lower limb tone and function sufficient to ambulate; however, recovery of contralateral upper limb function is not as frequently observed. In our series, 10/13 (76.9%) patients had an increased neurological deficit; however. these were expected, counselled for prior to surgery, and this accepted outcome reflected the severity of the child's epilepsy. Torres et al. [25] similarly assessed outcomes in 13 patients receiving PIH, noting that all patients had worsened hemiparesis at last follow-up. Neurological deficits were worsened in 28/68 patients (41.1%) in the systematic review; however, only 5 of the 13 studies described post-operative neurological deficits [7, 20, 24, 25, 29]. Furthermore, there is variability in these descriptions, where increased baseline deficits and new neurological deficits were not always demarcated. Large analyses describing neurological deficits post-hemispheric separation are few [6, 9, 15, 28]. Given the importance of this outcome to patients and families, it is clear that further assessment and standardization of post-operative neurological functioning following PIH is required.

Despite significant evolution in hemispherectomy techniques, hydrocephalus remains the most common complication of PIH [10, 15, 16, 33], as in other hemispherectomy techniques. Incomplete disconnection with ongoing seizures is another potential complication. The recording of complications other than hydrocephalus across the literature was not uniform in description and extent, and therefore analyses with respect to this are limited. The minimal sub-pial resection in PIH compared to other techniques may reduce rates of hydrocephalus [15, 30]; however, rates of VPS requirement after hemispherotomy are variable [6] and are likely modified by underlying aetiology. One comparative analysis of PIH and vertical hemispherotomy techniques described lower hydrocephalus rates (11.1%) for PIH compared to vertical approaches (21.1%) [16]. A large cohort analysis of 69 patients receiving PIH identified a hydrocephalus rate of 13.0% and overall complication rate of 27.8% [30] Our case-series observed 2/13 (15.4%) patients requiring post-operative VPS insertion for hydrocephalus, this being 21/244 (8.6%) patients in the systematic review and in keeping with broader hemispherotomy-combined reviews [15]. Greater need for VPS insertion was observed in the 2 studies with the youngest patients (average age 1.4 years) [6, 13], emphasizing the difference in benefit and risk balancing in very young patients.

### Limitations

The heterogenous nature of our pooled analysis from the systematic review delivers insights from a very large patient cohort which have good generalizability. However, this limits our findings as in many cases use of pre-operative investigations and some patient data were not available. Where possible, we have addressed elements of pre-operative workup such as EEG, PET, MRI, and demographic variables; however, this was not consistently possible. Furthermore, few studies described individualized patient data, and therefore subgroup analyses such as the comparison of developmental versus acquired pathology are achieved by a weighted proportion analysis for the study as a whole. Finally, five studies were class 3 evidence, the remainder class 4, highlighting a need for higher quality assessment of PIH outcomes.

## Conclusions

PIH uniquely approaches hemispheric separation from a lateral trans-insular plane. Differences in anatomical approach and patient selection between hemispherotomy techniques necessitate assessment of this technique in its own right. This systematic review and institutional series describes reassuring post-operative outcomes for patients suffering intractable epilepsy with over 4 in 5 patients having Engel 1 outcomes. Engel 1 outcomes are higher than reported in combined technique analyses, possibly due to insular resection and more complete disconnection. These outcomes are negatively impacted by developmental pathology; however, use of PIH in this context has similar outcomes to other hemispherotomy techniques, despite general preference of VPH for these patients. PIH in young patients may have significant seizure and cognitive benefits; however, a markedly increased risk of hydrocephalus warrants careful consideration of risks and benefits. The majority of analyses describing PIH outcomes and complications are class IV evidence, suggesting a need for more high-quality investigation. This review delivers a large independent analysis of PIH to support surgical decision-making and outcome counselling of future patients undergoing this procedure.

## Statement of Ethics

This study protocol was reviewed and approved by the Children's Health Queensland Human Research and Ethics Committee, Queensland (Registered with the National Health and Medical Research Council, #EC 00175, study approval number 1003230), in accordance with the Queensland Children's Hospital guidelines. Exemption from written informed consent from participants was granted by the Children's Health Queensland Human Research Ethics Committee (#EC 00175), Queensland. This research has been conducted in accordance with the guidelines for human studies and conducted ethically in accordance with the World Medical Association Declaration of Helsinki.

## Conflict of Interest Statement

All authors certify that they have no affiliations with or involvement in any organization or entity with any financial interest (such as honoraria; educational grants; participation in speakers' bureaus; membership, employment, consultancies, stock ownership, or other equity interest; and expert testimony or patent-licencing arrangements) or non-financial interest (such as personal or professional relationships, affiliations, knowledge, or beliefs) in the subject matter or materials discussed in the manuscript.

## Funding Sources

This research did not receive any specific grant from funding agencies in the public, commercial, or not-for-profit sectors.

## Author Contributions

All authors had substantial contributions to the conception and design of this study, review and interpretation of data, drafting the manuscript, and revising it critically for content. Charles Yates performed data collection and initial drafting of the manuscript. Martin Wood, Kate Riney, Stephen Malone, and Ubaid Shah were involved in patient care for the institutional cohort.

## Data Availability Statement

All data generated or analysed during this study are included in this article and its online supplementary material. Further enquiries can be directed to the corresponding author.

## Supplementary Material

Supplementary data

## Figures and Tables

**Table 1:** Systematic review of studies meeting eligibility criteria for inclusion, presenting demographic, clinical, and outcome patient data

Author	Study period	PIH patients, *n*	Evidence class	Age group	Gender (M/F)	Avg. age at op., years	Epilepsy duration, years	Cog. imp., %	Avg. follow-up, years	Engel 1, *n* (%)	Engel 1/2, *n* (%)	VPS	Mortality, *n* (%)	Neurological deficits
Kestle et al. [7]	1993–1999	11	4	Paediatric	NA	4.8	4.3	87.5	3.0	8/9 (88.9)	9/9 (100)	0/9	0/9	1/11 worse hemiparesis, 3/11 new hemianopia

Maehara et al. [20]	1994–1998	14	4	<6 years	8 M, 6 F	2.4	0.2	85.7	3.9	6/14 (42.8)	12/14 (85.7)	3/14 (21.4)	0/14	2/14 worse hemiparesis

Villemure and Daniel [5]	NA	43	3	Paediatric	25 M, 18 F	8.0	5.0	NA	9.0	34/37 (91.8)	NA	1/37 (2.7)	1/37 (2.7)	NA

Limbrick et al. [21]	2003–2008	35	3	Paediatric	21 M, 14 F	7.2	NA	NA	1.6	NA	28/30 (93)	NA	0/35	NA

Abraham et al. [22]	2005–2016	45	3	Paediatric	25 M, 20 F	8.0	5.2	NA	4.0	41/44 (93.2)	43/44 (97.7)	2/44 (4.5)	0/44	NA

Kumar et al. [23]	2002–2013	14	4	<1 year	NA	0.3	0.3	NA	5.3	11/14 (78.6)	12/14 (85.7)	4/14 (28.6)	1/14 (7.1)	NA

Granata et al. [24]	1993–2009	11	4	Adult/paediatric	4 M, 7 F	11.1	4.2	66.7	7.3	6/9 (66.7)	8/9 (88.9)	0/9 (0.0)	0/9	6/11 new hemianopia

Torres et al. [25]	2002–2007	13	4	Paediatric	9 M, 4 F	5.5	3.4	84.6	3.1	10/13 (76.9)	12/13 (92.3)	1/13 (7.7)	0/13	13/13 increased hemiparesis

Terra-Bustamante et al. [26]	1996–2005	16	4	Paediatric	13 M, 3 F	6.7	4.9	NA	1.9	10/16 (62.5)	12/16 (75)	NA	NA	NA

Kwan et al. [27]	1997–2007	20	3	Paediatric	11 M, 9 F	5.1	3.0	85.0	6.0	NA	17/20 (85)	1/20 (5.0)	0/20	NA

Arifin et al. [29]	1999–2019	19	4	Adult/paediatric	10 M, 9 F	13.3	2.6	NA	6.0	12/15 (80)	13/15 (86.7)	0/15 (0.0)	0/15	3/19 worse hemiparesis

Ji et al. [28]	2014–2017	83	3	Paediatric	55 M, 28 F	5.0	3.1	NA	3.0	69/83 (83.1)	70/83 (84.3)	NA	NA	NA

Weil et al. [30]	2000–2014	69	3	<19 years	44 M, 25 F	8.2	5.8	85.5	2.0	59/69 (81.2)	NA	9/69 (13.0)	0/69	NA

Weighted average		393			61.1% M	6.8	4.0	84.0	4.0	266/323 (82.4)	236/267 (88.4)	21/244 (8.6)	2/279 (0.7)	28/68 (41.1)

NA, not available; UTI, urinary tract infection.

**Table 2:** Aetiology in patients receiving PIH in the systematic review case series and relation to proportions with Engel class 1 outcomes

Author	Cohort date range	PIH patients, *n*	Acquired pathology		Developmental pathology		Engel 1 (%)
			total		total		
Kestle et al. [7]	1993–1999	11	1 (9.1)	RE (1)	10 (90.9)	CD (5), HME (2), PE (1), pachygyria (1), SWS (1)	88.9^a^
Maehara et al. [20]	1994–1998	14	0 (0)	–	14 (100)	CD (5), HME (8), polymicrogyria (1)	42.8^a^
Villemure and Daniel [5]	NA	43	36 (83.7)	Infantile hemiplegia (17), RE (13), ischaemic (2), infection (2), anoxia (1), trauma (1)	7 (16.3)	HME (3), migrational disorder (4)	91.8
Limbrick et al. [21]	2003–2008	35	–	NA	–	NA	–
Abraham et al. [22]	2005–2016	45	25 (55.6)	RE (11), ischaemic (6), infection (6), post-traumatic (2)	20 (44.4)	HHE (10), HME (4), CD (3), SWS (3)	93.2
Kumar et al. [23]	2002–2013	14	2 (14.3)	Vascular (1), gliosis (1)	12 (85.7)	HME (7), CD (4), SWS (1)	78.6^a^
Granata et al. [24]	1993–2009	11	11 (100)	RE (11)	0 (0)	–	66.7
Torres et al. [25]	2002–2007	13	6 (46.1)	RE (6)	7 (53.9)	CD (5), HME (2)	76.9^a^
Terra-Bustamante et al. [26]	1996–2005	16	10 (62.5)	RE (4), gliosis (4), PE (1), tumour (1)	6 (37.5)	CD (5), SWS (1)	62.5
Kwan et al. [27]	1997–2007	20	8 (40.0)	RE (8)	12 (60.)	HME (4), CD (7), SWS (1)	–
Arifin et al. [29]	1999–2019	19	–	NA	–	NA	80.0
Ji et al. [28]	2014–2017	83	57 (68.6)	RE (12), Parry Romberg syndrome (1), acquired unspecified (44)	26 (31.4)	HME (6), polymicrogyria (6), CD (5), pachygyria (4), heterotopia (2), SWS (3)	83.1
Weil et al. [30]	2000–2014	69	41/63 (65.1)	RE (13), ischaemic (28)	22/63 (34.9)	HME (11), CD (11)	81.2
		393		197/333 (59.2%)		136/333 (40.8%)	

NA, not available; HHE, hemiconvulsion-hemiplegia syndrome; HME, hemimegalencephaly; RE, Rasmussen encephalitis; SWS, Sturge-Weber syndrome; CD, cortical dysplasia; PE, porencephaly.

a Proportion of patients with developmental pathology is greater than 50%.

**Table 3:** Institutional series of paediatric patients receiving PIH from 2014 to 2020

No.	Gender	Age at diagnosis, years	Aetiology	Pre-op neurological deficit	Cog. imp	ILAE classification^a^	EEG lateralization	AEDs receiving pre-surgery, *n*	Age at op., years	Epilepsy duration, years	Engel score	Complications	Post-op. neurological deficit	AEDs post-op, *n*	Follow-up, years
1	Male	0.8	HHE	Right hemiplegia, right hemianopia	Yes	Focal; atonic, myoclonic	Left	2	5.8	5.0	2	Residual basal frontal connection requiring reoperation	Increased right leg weakness	1	2.5

2	Male	0.3	Ischaemic	Right hemiplegia, right hemianopia	Yes	Focal; tonic	Bilateral	3	2.1	1.8	1	Hydrocephalus - VPS	Unchanged	0	3.8

3	Male	1.7	Ischaemic	Right arm weakness, right hemianopia	Yes	Focal; tonic, epileptic spasms, CSWS on EEG	Bilateral	2	6.3	4.6	1		Increased hemiparesis	1	1.3

4	Female	10.0	RE	Right hemiplegia	Yes	Focal; epilepsy partialis continua	Left	2	13.7	3.7	1	–	Right hemiplegia, right hemianopia, dysphasia	0	1.0

5	Male	0.6	Ischaemic	Right hemiplegia, right hemianopia	Yes	Focal; tonic, atonic, absence^b^, CSWS on EEG	Bilateral	1	6.8	6.2	1	–	Unchanged	1	0.7

6	Male	0.3	SWS	Left hemiplegia, quadrantinopia	Yes	Focal non-motor, autonomic	Right	1	11.0	10.7	1	SIADH causing seizures	Increased left hemiplegia, left hemianopia	0	0.6

7	Male	8.0	Ischaemic	Left hemiplegia, left hemianopia	Yes	Focal non-motor, emotional	Right	1	10.1	2.1	1	Hydrocephalus - VPS	Unchanged	0	3.7

8	Female	1.1	SWS	Right hemiplegia, right hemianopia	Yes	Focal; clonic with impaired awareness Epileptic spasms	Bilateral	1	7.5	6.4	1	–	Increased right hemiplegia, right hemianopia	0	1.5

9	Male	0.2	CD	Right hemiplegia, right hemianopia	No	Focal; impaired awareness tonic, to bilateral, tonic-clonic Epileptic Spasms	Bilateral	4	0.9	0.7	1		Increased right hemiplegia, right hemianopia	0	1.1

10	Female	3.5	HHE	Left hemiplegia, left hemianopia	No	Focal; tonic	Right	2	5.9	2.4	1	Subdural peritoneal shunt	Left hemiplegia, left hemianopia, oculomotor nerve palsy	0	1.8

11	Female	0.9	HME	Left hemiplegia, left hemianopia	Yes	Focal; tonic to bilateral tonic-clonic	Bilateral	4	0.9	0	2	–	Left hemiplegia, right hemianopia	1	3.6

12	Male	0.5	CD	Right hemiplegia	No	Focal; tonic, automatism	Left	5	0.6	0.1	1	–	Increased right hemiplegia right hemianopia	3 (weaning)	0.5

13	Female	0.1	CD	Left hemiplegia Left hemianopia	Yes	Focal; tonic, epileptic spasms	Right	2	4.7	4.6	1		Increased left hemiplegia Left hemianopia	1 (weaning)	0.5

14	61.5% male	2.2	7/13 (53.8%; acquired		10/13 (76.9%)	3/13 (23.1%) bilateral spread		2.3	5.9	3.7	84.6% Engel 1	5/13 (38.5%)	10/13 (76.9%) increased deficit	0.6	1.7

HHE, hemiconvulsion-hemiplegia epilepsy syndrome; RE, Rasmussen encephalitis; SWS, Sturge-Weber syndrome; CD, cortical dysplasia; HME, hemimegalencephaly; VPS, ventriculoperitoneal shunt; SIADH, syndrome of inappropriate antidiuretic hormone; CSWS, continuous spike and wave during sleep.

a Where awareness is not reported, no impairment of awareness occurred.

b This patient experienced frequent absence/atypical seizures with generalized spike waves at 2–3 Hz on EEG as well as tonic and atonic seizures, suggesting evolution to a secondary network epilepsy [31, 32]; however, no generalized paroxysms of fast activity were observed in sleep.
